# Analysis of methyltransferase (MTase) domain from Zika virus (ZIKV)

**DOI:** 10.6026/97320630016229

**Published:** 2020-03-31

**Authors:** Sarah Afaq, Akhtar Atiya, Arshi Malik, Afaf S Alwabli, Dhafer A Alzahrani, Habeeb M Al-Solami, Othman Alzahrani, Qamre Alam, Mohammad Azhar Kamal, Aala A Abulfaraj, Alawiah M Alhebshi, Mohammed Tarique

**Affiliations:** 1 Department of Clinical Biochemistry, College of Medicine, King Khalid University, Abha, Kingdom of Saudi Arabia; 2 Department of Pharmacognosy, College of Pharmacy, King Khalid University, Abha, Kingdom of Saudi Arabia; 3 Department of Biological Sciences, Faculty of Science, King Abdulaziz University, Jeddah 21589, Kingdom of Saudi Arabia; 4 Department of Biology, Faculty of Science, University of Tabuk, Tabuk, Kingdom of Saudi Arabia; 5 Medical Genomics Research Department, King Abdullah International Medical Research Center, King Saud bin Abdulaziz University for Health Sciences, Ministry of National Guard Health Affairs, Riyadh, Kingdom of Saudi Arabia; 6 University of Jeddah, Faculty of Science, Department of Biochemistry, Jeddah, Kingdom of Saudi Arabia; 7 University of Jeddah Center for Science and Medical Research (UJC-SMR), Jeddah, Kingdom of Saudi Arabia; 8 Department of Biological Sciences, College of Sciences and Arts-Rabigh Campus, King Abdulaziz University, Jeddah 21589, Saudi Arabia; 9 Department of Biological Sciences, Faculty of Science, King Abdulaziz University, Jeddah 21589, Kingdom of Saudi Arabia; 10 Center for Interdisciplinary Research in Basic Sciences, Jamia Millia Islamia, Jamia Nagar, New Delhi-110025, India

**Keywords:** ZIKV, methyltransferase, beta turn, α-helix, SMART, Prosite, Pfam and InterProScan

## Abstract

A comprehensive analysis of methyltransferase (MTase) from Zika virus (ZIKV) is of interest in the development of drugs and biomarkers in the combat and care of ZIKA fever with impulsive joint pain and conjunctivitis. MTase sequence is homologous in several viral species. We analyzed the MTase domain from ZIKV using Bioinformatics tools such as SMART, PROSITE, PFAM, PANTHER, and InterProScan to glean insights on the sequence to structure to function data. We document inclusive information on MTase from ZIKV for application in the design of drugs and biomarkers to fight against the disease.

## Background

The Flavivirus Zika virus (ZIKV), which was announced as a Public Health Emergency by the World Health Organization (WHO) on February first, 2016. ZIKV is a virus from the Flaviviridae family and the Aedes mosquitoes acts as carriers [1-3]. The ZIKV genome is at first converted into a solitary precursor protein assembled into nonstructural proteins (1, 2A, 2B, 3, 4A, 4B and 5) [4]. The NS5 is the most significant and most monitored protein that contains methyltransferase (MTase) at the N-terminal region and an RNA-dependent RNA polymerase (RdRP) at C terminal region. It function in the replication of viral genome with RdRP and with methyltranferase domain separately [5]. Crystal structures of the MTase domain demonstrated that it possess the characteristic α/β overlay as found in the Dengue virus (DENV) [6], Japanese encephalitis (JEV) infection [7], or the ongoing structures of Zika virus (ZIKV) (5KQR) [8]. A sequence similarity among different flaviviruses is around 60%. The MTase domain NS5 protein consists of three subdomains. Initially, the C-terminal end has the conserved MTase crease framed by 7 strands β-sheet encompassed by 4 α-helices. In some structures an SAH (S-adenosyl-L-homocysteine) molecule is discovered bound to this domain [9]. The second sub-domain contains a helix-turn-helix theme, a β-strand and a α-helix structure at its N-terminals end. This domain was proposed to organize the GTP (guanosine-5′-triphosphate) moiety of 7-methylguanosine-GTP amid the 2'- O-ribose methylation as observed in the crystal structures bound to m7Gppp-RNA (7-methylguanosine cap at the 5' end of mRNA) [9]. The 3rd subdomain is situated between the two previous ones and is made out of an α-helix and two β strands [10]. Therefore, it is of interest to document broad information on MTase from ZIKV for application in the fight against the disease.

## Methodology:

### Sequence and conserved domain analysis MTase:

The sequence of the MTase domain was retrieved from the NCBI genome database followed by protein BLAST (BLASTp) analysis. The sequence was further subject to SMART, Prosite, Pfam, PANTHER, and InterProScan as described elsewhere [11-13].

### Analysis of predicted secondary structure:

The secondary structures were assigned using PSIPRED available at http://bioinf.cs.ucl.ac.uk/psipred.numbers refer to the amino acids sequence. In the box important active site amino acid highlighted in bold with different colors.

### Epitope prediction:

Epitope prediction was completed using the tool at http://tools.immuneepitope.org as described elsewhere [14].

### Structure analysis:

The sequence was further analyzed for structural features such as beta turns, helices and disallowed regions using tools as described elsewhere [15-19].

## Results and Discussion:

**Figure 1:**
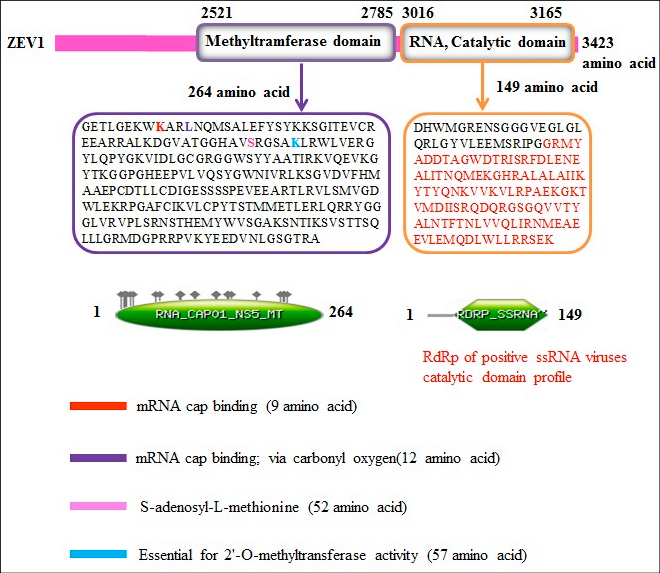
The detail domain organization of Zika virus (ZIKV). The conserved sequences of two important domains (MTase and RdRP) are written inside the boxes and highlighted. The text in purple and orange color box refers to the names of conserved domains and the numbers refer to the amino acids sequence. In the box important active site amino acid highlighted in bold with different colors.

**Figure 2:**
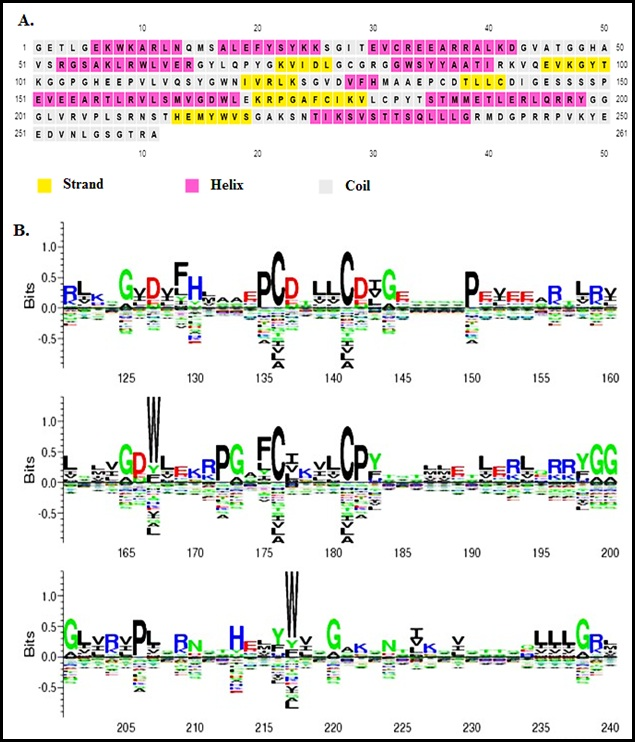
The protein sequences of MTase domain of Zika virus. A. The sequence was submitted to the server at http://bioinf.cs.ucl.ac.uk/psipred/. The graph represents the strand, helix and coil. B. Conservation of sequence within the specific MTase domain motifs. The peak of the amino acids residues reflects the level of retention at a position, and tall letters represents higher retention.

**Figure 3:**
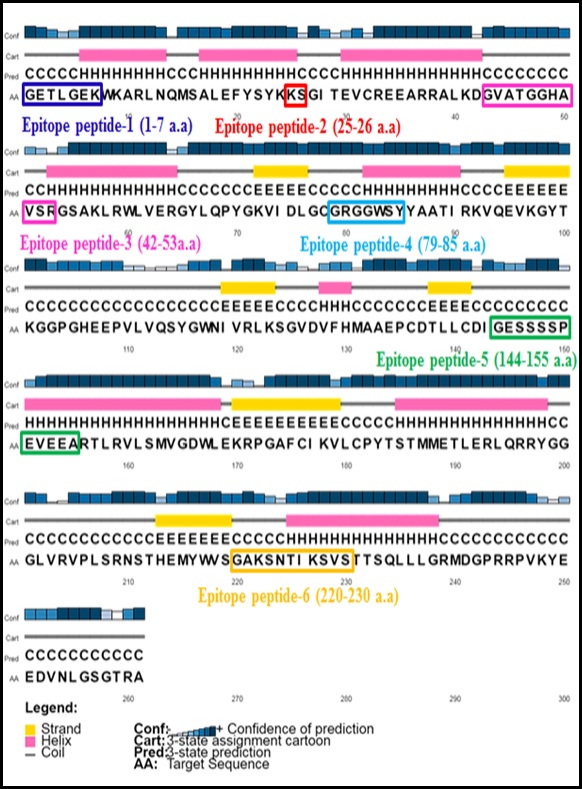
Prediction of structure (secondary) using server at http://bioinf.cs.ucl.ac.uk/psipred/. The protein sequence of MTase domain of Zika virus was submitted to the server and the secondary structures was determined. The graph represents the structures of MTase domain of Zika virus. Epitope peptide (6) boxed in different color the numbers refer to the amino acids sequence.

The Sequence analysis and domain organization of MTAse domain and (264 amino acids) and RdRp area (149 amino acids) is shown using SMART, Prosite, Pfam, PANTHER, and InterProScan in (Figure 1). There is three crucial amino acid arrangement of MTase domain are in charge of dynamic site restricting which are mRNA top official (K), mRNA top authoritative; using of carbonyl oxygen (L), S-adenosyl-L-methionine (S) and Essential for 2'- O-methyltranferase action (K). Amino acid consensus logo based analysis of the MTase domain with strands, α-helix, and coil is shown in (Figure 2A). Different residues at the same location are scaled on the basis of residue frequency as shown in (Figure 2B). Secondary structure and antigenic determinant of the MTase domain is shown in (Figure 3). The major epitope peptides are six that are highlighted in color boxes (Figure 3). Data on beta turns in the MTase is given in Table 1 and Table 2. Data on helices in the MTase domain is given in Table 3. Thus, we document inclusive information on MTase from ZIKV for application through a comprehensive understanding in the design of drugs and biomarkers to fight against the disease caused by the virus.

## Conclusions:

We document prelimianary information from a comprehensive analysis on MTase from ZIKV using Bioinformatics tools such as SMART, PROSITE, PFAM, PANTHER, and InterProScan to glean insights on the sequence to structure to function data for combat and care of ZIKA fever.

## Figures and Tables

**Table 1:** The turns are doled out to one of 9 classes based on the phi, psi edges of buildups i+1 and i+2. The perfect plots for every one of the turn types are as per the following:

Type	Phi(i+1)	Psi(i+1)	Phi(i+2)	Psi(i+2)	
I	-60	-30	-90	0	
II	-60	120	80	0	
VIII	-60	-30	-120	120	
I’	60	30	90	0	
II’	60	-120	-80	0	
VIa1	-60	120	-90	0	cis-proline(i+2)
VIa2	-120	120	-60	0	cis-proline(i+2)
VIb	-132	135	-75	160	cis-proline(i+2)
IV	Turns excluded from all the above categories				

**Table 2:** Beta turns in the MTase domain from ZIKV

No.Turn	Sequence*	Turn Type	Residue i+1			Residue i+2			I to i+3	H
			Phi	Psi	Chi1	Phi	Psi	Chi1	CA-dist	bond
1.*Lys29-IIe32	KSGI	II	-64.5	116.6	-77.1	70.6	8.5	-	5.5	No
2.*Val48-Gly51	VATG	IV	-110.1	-6.4	-	-116.6	15.2	55.6	6.4	Yes
3.*Gly69-Gln72	GYLQ	VIII	-72.8	-18.3	-58.3	-143.9	132.4	171.2	6	Yes
4.*Asp79-Cys82	DLGC	IV	-90.9	125.2	-59.7	74.3	53.6	-	5.6	No
5.*Cys82-Gly85	CGRG	II	57	-134.9	-	-64.3	-27.5	-66	5	No
6Ile94-Val97	IRKV	I	-56	-44.2	-173	-75.7	-13.8	-73.5	5.4	No
7Gly107-His110	GPGH	II	-56	126.3	-30.7	83.1	5.4	-	5.6	No
8Ser118-Trp121	SYGW	II	59.8	128.4	-178.6	85.9	11.5	-	5.6	No
9Lys127-Val130	KSGV	IV	-96.1	173.4	-168.8	71.5	24.4	-	6.9	Yes
10Cys140-Leu143	CDTL	VIII	-93.2	-37.6	-58.9	-128.1	124.9	-56.8	6.7	Yes
11Ser151-Pro154	SSSP	VIII	-68.7	-16.9	57.6	-89.7	119.5	162.8	6.9	Yes
12Pro176-Phe154	PGAF	VIII	-84.4	-19.9	-	-129.7	143.1	-	6.9	Yes
13Val183-Pro186	VLCP	VIII	-59.7	-46.5	169	-129.5	91.6	175.5	5.9	Yes
14Val209-Ser212	VPLS	I	-68.9	-13.5	19	-75.5	-11.7	-63.1	5.7	No
15Arg213-Thr216	RNST	I	-66.4	-14.5	-62.1	-82.5	-0.9	51	6	No
16Val222a225r223-Lys226	VSGA	I	-71.1	-23.4	45.3	-95.8	8.6	-	6	No
17	SGAK	IV	-95.8	8.6	-	-42	75.8	-	6.9	Yes

**Table 3:** Helices in the MTase domain from ZIKV

No.	Start	End	Type	No. Resid	Length	Unit Rise	Residue Per turn	Pitch	Deviation from Ideal	Sequence
1.*	Leu8	Asn17	H	10	15.63	1.51	3.59	5.43	2.2	LGEKWKARLN
2.*	Ala21	Lys28	H	8	12.52	1.48	3.79	5.6	13.5	ALEFYSYK
3.*	Glu38	Lys45	H	8	12.47	1.51	3.71	5.6	11.9	EEARRALK
4.*	Gly58	Glu67	H	10	15.72	1.5	3.69	5.54	8.8	GSAKLRWLVE
5	Gly86	Ala92	H	7	11.28	1.54	3.65	5.35	7.2	GWSYYAA
6	Trp121	Ile123	G	3	-	-	-	-	-	WNI
7	Val132	His134	G	3	-	-	-	-	-	VFH
8	Pro154	Leu172	H	19	28.29	1.46	3.66	5.35	13.4	PEVEEARTLRVLSMVGDWL
9	Ser189	Tyr202	H	14	20.03	1.45	3.6	5.23	9.7	STMMETLERLQRRY
10	Thr229	Gly242	H	14	21.41	1.49	3.69	5.49	4.1	TIKSVSTTSQLLLG

**Figure 4:**
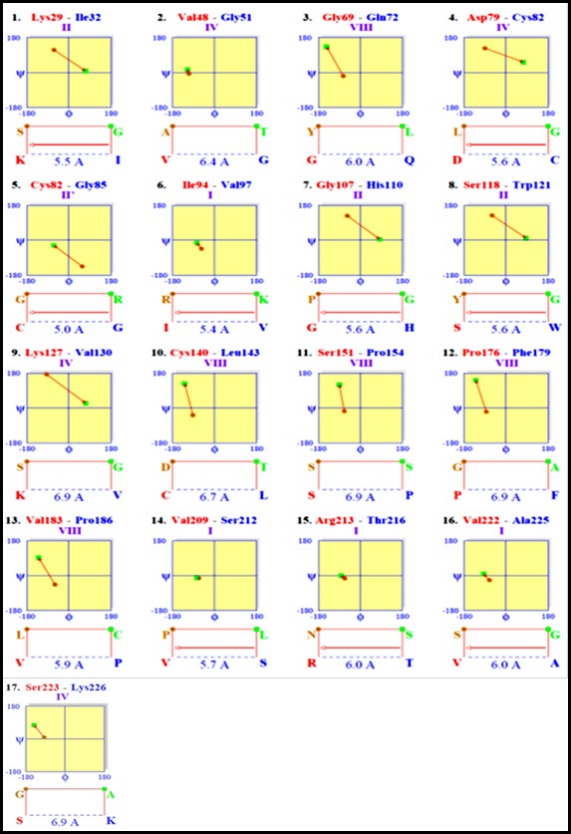
The plots for turns demonstrate a Ramachandaran plot with residues i+1 (brown circle) and i+2 (green square) plotted on it. The following is a graphic plot of the turn with the four amino acid residues and marked C alpha (i) C alpha (i+3) distance. A red arrow, if present, indicates that residue I donate a hydrogen bond to residue i+3. The numbers of residue and type of turn are demonstrated over the Ramachandaran plot.

**Figure 5:**
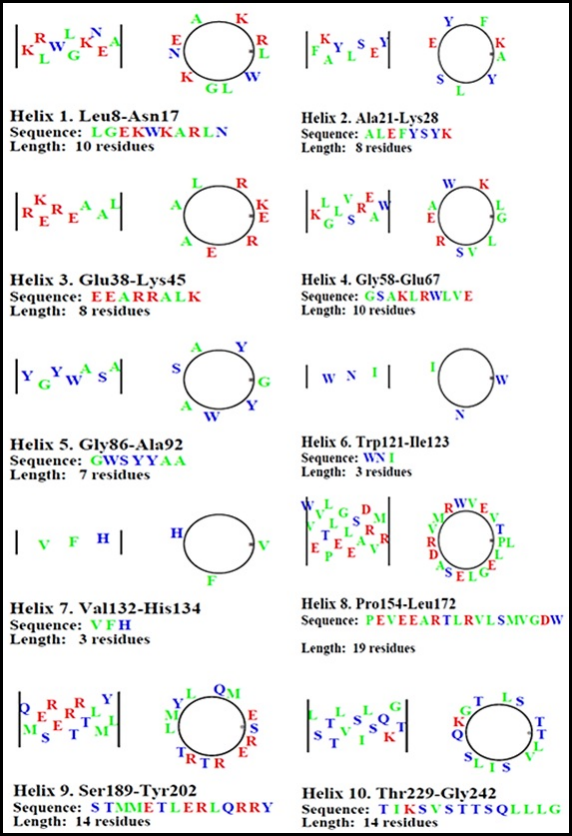
The Helical haggle, and net' color diagrams represent the organization of the amino acid residues in every helix. The amino acid residues are in green color for hydrophobic, blue color for polar and red color for charged amino acid. Haggles and nets accepted the helical estimation of 3.6 residues per turn.
